# Effects of empagliflozin on cyclophosphamide-induced neurotoxicity in rats without diabetes

**DOI:** 10.55730/1300-0144.5943

**Published:** 2024-12-30

**Authors:** Şerife Ezgi DOĞAN, Şerife Mehlika KUŞKONMAZ, Pınar CELEPLİ, Mehmet ŞENES, Sema HÜCÜMENOĞLU, Cavit ÇULHA

**Affiliations:** 1 Division of Endocrinology, Department of Internal Medicine Ankara Training and Research Hospital, Ankara, Turkiye; 2 Department of Pathology, Ankara Training and Research Hospital, Ankara, Turkiye; 3 Department of Biochemistry, Ankara Training and Research Hospital, Ankara, Turkiye

**Keywords:** Cyclophosphamide neurotoxicity, rat brain, empagliflozin

## Abstract

**Background/aim:**

The objective of this study was to evaluate the effects of empagliflozin on cyclophosphamide-induced neurotoxicity in rats.

**Materials and methods:**

A total of 32 male Wistar rats were separated into 4 groups with 8 rats in each: the control group, cyclophosphamide group, empagliflozin group, and cyclophosphamide plus empagliflozin group. At the end of the experiment the rats’ brains were removed for biochemical analysis of oxidative stress parameters (malondialdehyde, total sulfhydryl, total oxidant status, and total antioxidant capacity) and histopathological analysis.

**Results:**

Total sulfhydryl levels were increased in the empagliflozin group and empagliflozin and cyclophosphamide group compared to the cyclophosphamide group but histopathological findings were not improved. Furthermore, when empagliflozin treatment was used coupled with cyclophosphamide, necrosis was significantly higher.

**Conclusion:**

Empagliflozin has no protective effect on cyclophosphamide neurotoxicity, although it may have neurotoxic effects. Clarification of the possible neurotoxic/neuroprotective effects of empagliflozin would be beneficial for diabetic cancer patients who are receiving cyclophosphamide chemotherapy and empagliflozin together.

## 1. Introduction

Sodium glucose-linked cotransporter 2 (SGLT2) inhibitors are antihyperglycemic drugs that mainly target SGLT2 in the proximal tubule of the kidney and increase glucose excretion. These agents are not wholly SGLT2 selective and have an affinity for SGLT1 [1]. In addition to the kidneys, sodium glucose-linked cotransporters have been demonstrated in many organs, including brain tissue [1,2]. SGLT2 inhibitors are lipid soluble and cross the blood–brain barrier [3]. Numerous reports have demonstrated the presence of SGLT2 and SGLT1 receptors in the mammalian brain, which suggests their potential role in the regulation of neuronal activity [4,5]. It can directly affect their target in the human brain and potential role in the regulation of glucose homeostasis [6]. They are safe medications with manageable adverse effects including genital mycotic infections and volume depletion [7].

Empagliflozin is a selective SGLT2 inhibitor. Its cardiorenal benefits are explained by the induction of the fasting-like metabolic switch, which includes low insulin levels, downregulation of glycolysis, and a decrease in inflammation [8]. Recent preclinical studies show that empagliflozin may ameliorate common brain pathologies via oxidative stress and inflammatory pathways. In animal studies, it has been observed that empagliflozin reduces the production of oxidative stress parameters and inflammatory mediators in the brain of hyperglycemic mice, which was associated with improvement in cognitive function and cerebral ischemia/reperfusion damage reduction [9,10]. In addition, some studies demonstrate these effects in a dose-dependent manner [9]. On the other hand, an in vitro study exhibited toxic effects in higher concentrations of empagliflozin.^1^ Cyclophosphamide (CYP) is a chemotherapeutic drug. Its mechanism interrupts DNA replication in rapidly dividing cancer cells. However, CYP influences healthy organs including the brain and leads to side effects like chemobrain, nausea, and emesis [11]. CYP neurotoxicity occurs due to oxidative inflammatory cascades and apoptotic signaling in the molecular mechanism. Depletion of the cerebral antioxidant mechanism implicates oxidative stress and gives rise to cellular impairments, such as massive cellular degeneration, proinflammation, and apoptosis in rats’ brains [12,13]. The present study aimed to investigate the potential effects of empagliflozin on CYP-induced neurotoxicity in rats.

## 2. Materials and methods

### 2.1. Drugs and chemicals

The cyclophosphamide (Endoxan, Baxter) was reconstituted by adding saline (vial of 500 mg with 25 mL of saline). The vial was shaken vigorously for several minutes until complete dissolution of the powder. Before use, the vial was checked to see whether the mixture was clear and colorless and it was given to the rats 100 mg/kg/week intraperitoneally (IP) (the final concentration was 20 mg in each milliliter of 0.9% sodium chloride).

The empagliflozin (Jardiance, Boehringer Ingelheim) was dissolved and diluted with saline (25-mg film-coated tablets with 25 mL of saline) and administered to the rats 10 mg/kg/day by oral gavage (the final concentration was 1 mg in each milliliter of 0.9% sodium chloride).

### 2.2. Animals and experimental protocol

Thirty-two healthy male Wistar albino rats weighing between 250 and 300 g and aged 24 weeks were used. The rats were kept at 20–21 °C and 50 ± 10% humidity in a 12-h light/dark cycle and had free access to food and water until 2 h before the anesthesia procedure.

The rats were randomly divided into four groups (n = 8/group). For 4 weeks, the CYP group animals were administered cyclophosphamide IP at 100 mg/kg per week, the EMPA group animals were administered by oral gavage empagliflozin 10 mg/kg once daily, the CYP + EMPA group animals were administered cyclophosphamide IP at 100 mg/kg per week and with empagliflozin 10 mg/kg by oral gavage once daily, and the control group animals were administered 0.9% saline IP once every week [12,14].

At the end of the experiment, all the rats were anesthetized by ketamine injection (0.5 mL/kg ketamine IP) and the cervical artery was cut to obtain exsanguination. The brains were removed from each rat and the cerebral cortex was cut for examination. The histopathological changes in neurons were evaluated.

### 2.3. Biochemical analysis of oxidative stress parameters

Oxidative stress parameters, i.e. malondialdehyde (MDA), total sulfhydryl (T-SH) levels, total oxidant status (TOS), and total antioxidant capacity (TAC), were analyzed in the brain tissue of the rats in the Biochemistry Department of Ankara Education Training and Research Hospital. The brain tissue samples were stored at −80 °C until analysis and homogenized with 0.05 M PBS (pH 7.4) at a rate of 1/10 (weight per volume). The homogenate was centrifuged at 3000 × *g* at 4 °C for 10 min. The supernatant was used for the biochemical analyses. MDA, T-SH, TOS, and TAC levels were measured in the tissue samples [15,16].

MDA levels were measured using the spectrofluorometric method (Hitachi F-2500 spectrofluorometer; Berkshire, UK) and T-SH levels were determined with the spectrophotometric method (Shimadzu CL-770 spectrophotometer; Shimadzu Scientific Instruments, USA). TAC and TOS measurements were performed in a Cobas c501 analyzer with automated methods using the Rel Assay Diagnostics kits (Turkey) as described by Erel [17,18].

### 2.4. Histological analysis

Brain tissue samples taken from rats were fixed in 10% formaldehyde solution for 48 h, followed by ethanol dehydration (50%, 75%, 96%, and 100% consecutively) and xylene transparency, and then embedded in paraffin. Next 4-μm sections were taken from the paraffin-embedded tissues with a Leica RM 2125 RT microtome. The sections of tissues were stained using hematoxylin and eosin (H&E) and then analyzed. The histopathological examination was conducted by a blinded pathologist using an Olympus BX51TF model at 4×, 10×, 20×, 40×, and 100× magnifications. Edema, congestion, degeneration, inflammation, necrobiosis, and necrosis in the H&E stained sections were evaluated using a semiquantitative scoring system. The scoring systems were as follows: edema, congestion, and degeneration: none (0), mild (1), severe (2); necrobiosis/necrosis and inflammation: no (0), yes (1) (Table 1) [12,19].

### 2.5. Statistical analysis

The Shapiro–Wilk test was used to determine whether the distribution was normal. Normally distributed data were reported using mean±standard deviation (SD) and nonnormally distributed data using descriptive statistical methods such as median (min–max). A comparison of continuous variables was performed using one-way ANOVA for normally distributed data and the Kruskal–Wallis test for nonnormally distributed data. The differences between the continuous variables were evaluated by two-tailed t-test. All statistical analyses were performed using SPSS Statistics version 23.0 (IBM Corporation, Armonk, NY, USA). P-values less than 0.05 value was considered statistically significant. If p-values were significant in the first tests, the post hoc Tukey HSD test or Mann–Whitney U multiple comparison test was applied to determine from which group the difference originated.

### 2.6. Ethical issues

All procedures in the study were applied in compliance with the National Guidelines for the Use and Care of Laboratory Animals (Türkiye). Approval for the study was granted by the Local Ethics Committee of Ankara Education and Research Hospital, Türkiye (protocol no: 0046, dated: 15.01.2020).

## 3. Results

Three rats in the CYP group, one rat in the CYP + EMPA group, and one rat in the EMPA group died at the beginning of week 4.

### 3.1. Biochemical analysis of oxidative stress parameters

Table 2 shows the effect of EMPA and CYP on cerebral markers of oxidative stress.

CYP injection provoked significant depression of SH and TAC levels and an increase in MDA in comparison to the control. On the other hand, cerebral T-SH levels were significantly increased in the EMPA and CYP + EMPA groups compared to group CYP. The CYP + EMPA group had lower MDA levels compared to the CYP group but the difference was not significant. No difference was observed in the levels of TOS between the groups.

### 3.2. Histopathological findings

**Figure 1:**
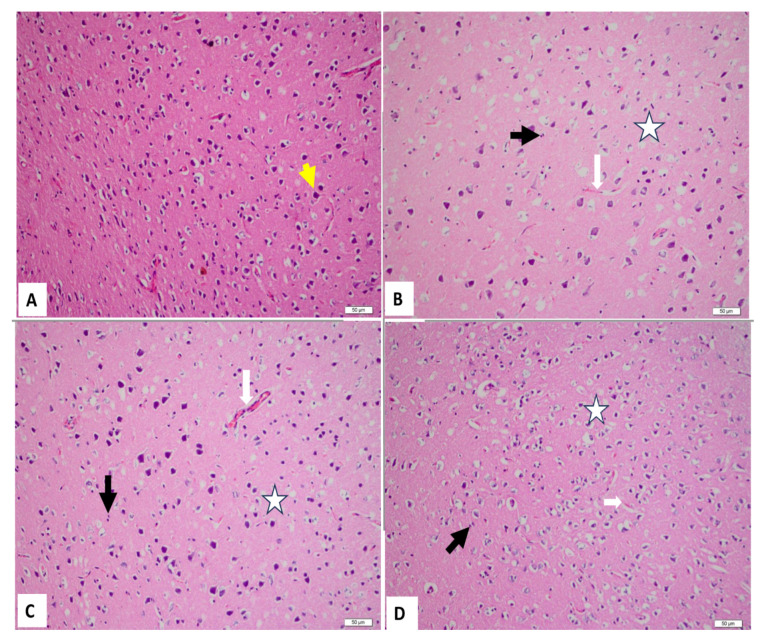
Light microscopy of brain tissues in different groups. A. Normal neuronal cells (yellow arrow) were observed in the cerebrum samples of the control group (H&E, 200×). B. CYP injection resulted in edema, congestion (white arrow), degeneration (star), and inflammation (black arrow) (H&E, 200×). C. The EMPA group showed edema, degeneration (star), and inflammation (black arrow) when compared to the control group (H&E, 200×). D. Edema, congestion (white arrow), inflammation (black arrow), degeneration (star), and necrobiosis were also demonstrated in the CYP + EMPA group (H&E, 200×).

The histopathological examinations of the study groups stained with H&E are shown in Table 3 and Figure 1. Normal neuronal cells were observed in the cerebrum samples of the control group (Figure 1a). CYP injection resulted in edema, congestion, degeneration, and inflammation (Figure 1b), whereas rats in the EMPA group showed edema, degeneration, and inflammation (Figure 1c) when compared to the control group. Additionally, necrobiosis and necrosis were also demonstrated in the CYP + EMPA group (Figure 1d). No significant difference was detected between the other groups.

## 4. Discussion

We investigated the effects of empagliflozin on CYP neurotoxicity in rats’ brains by evaluating the oxidative stress parameters and light microscopic assessment. Compatible with previous studies, the imbalance of oxidant/antioxidants favored oxidants and pathological findings of neurotoxicity were observed with CYP injection in the brains of the rats. Empagliflozin treatment increased the T-SH level compared to the CYP treatment but did not improve histopathological findings.

Oxidative stress is the leading component of CYP-incited neurotoxicity [12]. Oxygen-containing reactive species may attack polyunsaturated fatty acids, causing lipid peroxidation and MDA formation [20]. Consistent with previous studies, cerebral MDA levels increased after CYP treatment according to our results [21,22]. In addition, we assessed the T-SH levels as this is straightforwardly related to enhancement of the brain against oxidative stress. Glutathione is the main antioxidant apparatus containing sulfhydryl and CYP creates oxidative stress by diminishing glutathione levels owing to its hepatic metabolite acrolein [23]. In agreement with these outcomes, T-SH levels were reduced by CYP treatment in the current study.

Cerebral TAC levels decreased with CYP in the present research. Salimnejad et al. reported diminished TAC levels in the epididymides of rats after intraperitoneal CYP injection and negative effects on fertility rates [24]. The disturbance in antioxidant defense systems including TAC may contribute to CYP neurotoxicity like genotoxicity.

In the present study, we did not see significant changes in cerebral TOS levels. A few reports have exhibited rising serum TOS levels after CYP injection but no study has assessed cerebral TOS levels [25]. In contrast, Mert et al. demonstrated elevated cerebral TOS levels after intraperitoneal cisplatin [26]. To evaluate CYP neurotoxicity, it may be more appropriate to examine the brain and serum levels of TOS simultaneously.

The changes in cerebral MDA, T-SH, and TAC levels may affect the mechanism of CYP neurotoxicity, consistent with previous reports. Cerebral TOS levels can be investigated in future studies on whether there was a difference in CYP neurotoxicity.

Oxidative stress triggers inflammatory cascades [21]. The induced inflammation caused histopathological lesions such as inflammatory cells, degeneration, and congestion [27]. The results of the current study agree with the observations of past studies that postulated that CYP caused highly significant dystrophic alterations in the neurocytes [12]. SGLT2 inhibitors were demonstrated to ameliorate oxidative stress, not only by preserving a normal glucose level but also by reducing the generation of free radicals [28]. Empagliflozin treatment limits oxidative stress parameters in the hyperglycemic rat’s brain [14]. Furthermore, the same outcomes were demonstrated in studies that evaluated the effects of oxidative stress in nondiabetic rats. In our research, when empagliflozin treatment was used together with CYP, it decreased MDA levels, but no significant difference was found. However, empagliflozin treatment increased T-SH levels compared to the CYP group and CYP + EMPA group in concordance with studies describing antioxidant effects in rats without hyperglycemia [9,29].

In experimental studies, empagliflozin attenuates inflammation in nondiabetic mice [29,30]. However, empagliflozin administration did not reduce CYP-induced inflammation or degeneration but it increased inflammatory cells in the current examination. Furthermore, when empagliflozin treatment was used coupled with CYP, necrosis was significantly higher.

This hypothesis was in line with an investigation that denoted the toxic effects of empagliflozin in primary cell cultures. Primary neuron cells were obtained from nondiabetic rats’ brains exposed to different empagliflozin doses and higher concentrations of empagliflozin exerted toxic effects in the aforementioned report.^1^ In addition, Ahmed et al. observed improvements in neurodegenerative findings with low-dose (one fifth of our dose) empagliflozin in rotenone-induced Parkinson’s disease in mice [9].

On the other hand, Amer et al. showed that a similar empagliflozin dose reduced vigabatrin cerebellar toxicity due to the adenosine monophosphate-activated protein kinase (AMPK)/mammalian target of rapamycin (mTOR) signaling pathway. Their report demonstrated empagliflozin provoked AMPK and reduced mTOR levels compared to vigabatrin [29]. In another report, CYP increased cognitive dysfunction via AKT signaling, which stimulates the mTOR pathway [31]. Empagliflozin may exert its effects in the brain mostly through the AMP kinase/mTOR pathway. This may have reduced the effect of empagliflozin on CYP neurotoxicity, which stimulates the mTOR pathway through AKT activity. The role of the mTOR pathway in CYP neurotoxicity needs to be explored in future studies.

Our study has several limitations. The first is the fact that proinflammatory cytokine levels of interleukin-1β, interleukin-6, and tumor necrosis factor-alpha were not measured. These parameters could provide a more objective assessment of inflammation. Second, we could not perform immunohistochemical staining due to technical deficiencies and inability. Essentially glial fibrillary acidic protein and caspase-3 will be helpful to demonstrate the action of neurotoxicity and apoptosis. The third limitation is the absence of renal function, liver function and serum albumin levels at the end of study due to technical deficiencies. Further proof is needed to evaluate the effects of empagliflozin on the brain.

We conclude that empagliflozin treatment is not protective against cyclophosphamide neurotoxicity. In addition, studies have displayed mTOR effects of empagliflozin by AMPK and CYP by AKT pathways to date. Therefore, new studies are planned to employ different doses of empagliflozin and target signaling pathways to evaluate its effect on CYP neurotoxicity. Clarification of the possible neurotoxic/neuroprotective effects of empagliflozin would be beneficial for diabetic cancer patients who are receiving CYP chemotherapy and empagliflozin together.

## Figures and Tables

**Table 1:** The histopathological assessment scale.

	0	1	2
Necrobiosis/necrosis	No	Yes	
Inflammation	No	Yes	
Edema	No	Mild	Severe
Congestion	No	Mild	Severe
Degeneration	No	Mild	Severe

**Table 2:** Effects of EMPA and CYP on oxidative stress parameters in rats.

	Group Control^a^ (n = 8)	Group CYP^b^ (n = 5)	Group EMPA^c^ (n = 7)	Group CYP + EMPA^d^ (n = 7)	p

MDA (nmol/g protein), mean ± SD	771 ± 226.5^b^	1033 ± 130^a^	906 ± 87.6	862 ± 115.2	0.03^a^,^b^

T-SH (μmol/g protein), mean ± SD	134.3^b^ (103.7–189.0)	57.2^a^,^c^,^d^ (55.5–103.1)	154.9^b^ (119.7–181.0)	159.2^b^ (88.1–171.3)	0.007^a^,^b^
					0.003^b^,^c^
					0.01^b^,^d^

TAC (μmol/g protein), mean ± SD	123.4^b^, (103.7–174.4)	82.5^a^ (58.1–99.5)	121.2 (37.0–137.6)	86.5 (37.2–352.5)	0.007^a^,^b^

TOS (μmol/g protein), mean ± SD	3.8 (1.0–10.6)	4.8 (3.1–6.6)	2.6 (0.52–7.11)	1.8 (1.3–9.9)	0.38

Abbreviations: MDA: Malondialdehyde, SD: standard deviation, TAC: total antioxidant capacity, TOS: total oxidant status, T-SH: Total sulfhydryl

Data with normal distribution (MDA) were presented as mean ± SD and data without normal distribution (T-SH, TAC, TOS) were presented as median (min–max). Comparison of continuous variables was performed using one-way ANOVA with Tukey’s post hoc test for normally distributed data and the Kruskal–Wallis test for nonnormally distributed data.

a Denotes statistical significance (p < 0.05) when compared to control group.

b Denotes statistical significance (p < 0.05) when compared to CYP group.

c Denotes statistical significance (p < 0.05) when compared to EMPA group.

d Denotes statistical significance (p < 0.05) when compared to CYP + EMPA group

**Table 3:** Brain tissue histopathological evaluation of groups.

	Group Control (n = 8)	Group CYP (n = 5)	Group EMPA (n = 7)	Group CYP + EMPA (n = 7)	p value
Edema (0.00–2.00)	0.25 (0.00–1.00)	2.40 (2.00–3.00)	2.42 (2.00–3.00)	2.71 (2.00–3.00)	**<0.001** **0.025**^a^, **0.008**^b^, **0.001**^c^, 1.00^d^ 1.00^e^, 1.00^f^
Congestion(0.00–2.00)	0.00 (0.00–0.00)	3.00 (3.00–3.00)	2.28 (2.00–3.00)	2.85 (2.00–3.00)	**<0.001** **<0.001**^a^, 0.063^b^ **<0.001**^c^, 0.683^d^,1.00^e^, 0.995^f^
Degeneration (0.00–3.00)	0.00 (0.00–0.00)	2.8 (2.00–3.00)	1.71 (1.00–3.00)	2.57 (2.00–3.00)	**<0.000** **0.001**^a^**,0.014**^b^, **0.000**^c^, 1.00^d^, 1.00^e^, 1.00^f^
Necrobiosis/necrosis (0.00–1.00)	0.00 (0.00–0.00)	0.4 (0.00–1.00)	0.14 (0.00–1.00)	0.71 (0.00–1.00)	**0.019** 0.79^a^, 1.00^b^, **0.018**^c^, 1.00^d^, 1.00^e^, 0.130^f^
Inflammation (0.00–1.00)	0.00 (0.00–0.00)	1.00 (1.00–1.00)	0.85 (0.00–1.00)	1.00 (1.00–1.00)	**<0.001** **0.002**^a^, **0.003**^b^,**<0.001**^c^, 1.00^d^,1.00^e^, 1.00^f^

a Significance comparison between control and CYP groups.

b Significance comparison between control and EMPA groups.

c significance comparison between control and CYP + EMPA groups.

d Significance comparison between CYP and EMPA groups.

e Significance comparison between CYP and CYP + EMPA groups.

f Significance comparison between EMPA and CYP + EMPA groups.
